# Colonial Mentality and Diabetes Self‐Management in Filipino Americans

**DOI:** 10.1002/nop2.70175

**Published:** 2025-02-27

**Authors:** Rey Paolo Ernesto Roca, Behnan Albahsahli, Gilkevyn Joseph Gaw Palao, Gillkaitlyn Mary Gaw Palao, Derek Lance, Katrina Rae Carpizo, Dante Anthony Tolentino

**Affiliations:** ^1^ University of California Los Angeles California USA; ^2^ University of California San Diego California USA; ^3^ University of Michigan Ann Arbor Michigan USA

## Abstract

**Aim:**

To examine the correlation between colonial mentality and diabetes self‐management among Filipino Americans with T2DM.

**Design:**

Cross‐sectional descriptive and correlational survey study.

**Methods:**

This cross‐sectional study used the *Colonial Mentality Scale for Filipino Americans* to measure colonial mentality and the *Diabetes Self‐Management Questionnaire* to measure self‐management behaviours. The online survey was administered to Filipino Americans aged 18 or older with type 2 diabetes who resided in the United States. Recruitment was conducted from 2022 to 2023 through social media and Filipino American organisations. Data from 37 participants were analysed using descriptive statistics, an independent *t*‐test, and Pearson's *r* correlation.

**Results:**

There was a statistically negative moderate association between two manifestations of colonial mentality (internalised cultural/ethnic inferiority and cultural shame) and several diabetes self‐management behaviours: glucose control, healthcare use, and overall self‐management. Colonial mentality manifestations were greater in males than in females, although not statistically significant. This is the first study to empirically examine the association between colonial mentality and diabetes self‐management in Filipino Americans.

## 1 Introduction

Filipino Americans are the third‐largest Asian American ethnic group in the United States; however, they remain understudied in research and are often referred to as the forgotten minority (David 2016; Cordova et al. 1983). Despite Filipino Americans having positive economic indicators, they have a higher proportion of poor health and higher rates of chronic diseases such as diabetes and hypertension compared to other Asian American groups (Adia et al. 2020). Filipino American scholars posit that colonial mentality, a type of internalised racism rooted in colonialism, is intertwined with Filipino Americans' experiences of health inequities, which impact how Filipino Americans manage their health (Sabado‐Liwag et al. 2022). While we have long understood the influence of social determinants of health (SDOH) on health outcomes, the racial and colonial hierarchies that underpin these SDOH remain underacknowledged and inadequately addressed (Social and Community Context—Healthy People 2030 | health.gov n.d.). Structural racism, including systemic inequities in healthcare and social systems, has been linked to poorer outcomes, suboptimal standards of care, and disparities in diabetes self‐management behaviours, particularly among marginalised groups (Egede et al. 2023).

## 2 Background

### 2.1 Colonialism and Colonial Mentality

Colonialism is the process by which one country exerts control over another, often through political, economic, and cultural domination (Sabado‐Liwag et al. 2022). Filipino Americans are the only Asian American ethnic group that has experienced direct colonisation by the United States (Tuazon et al. 2019), with the Philippines' long history of colonisation contributing to the internalisation of colonialism among Filipinos (David and Okazaki 2006). Rooted in colonialism, colonial mentality involves the internalisation of beliefs that shape individual and societal attitudes about cultural identity. Even after independence from Spanish and American rule, colonial mentality continues to overtly or covertly manifest in Filipino culture (Decena 2014).Colonial mentality is described as having a sense of cultural shame (i.e., denigration of the Filipino self), feelings of cultural inferiority, colonial debt (i.e., tolerance of historical and contemporary oppression), and within‐group discrimination (e.g., discrimination against less‐Americanized Filipinos) (Sabado‐Liwag et al. 2022; Tuazon et al. 2019; David and Okazaki 2006; Brown David 2013; David and Nadal 2013). In short, it is characterised by a deferential attitude toward Western culture and the devaluation of Filipino culture (Brown David 2013). As colonial mentality is a consequence of colonialism and contemporary oppression that persists after colonisation, it is an important construct to consider when examining Filipino Americans' experiences of health inequities (David and Nadal 2013).

### 2.2 Type 2 Diabetes and Cultural Aspects of Diabetes Self‐Management

Type 2 diabetes mellitus (T2DM) prevalence in Filipino Americans is 10%–16%, significantly higher than in non‐Hispanic Whites (7%) (Raquinio et al. 2021). They have the second highest prevalence rate among Asian Americans and are almost three times at a greater risk of having T2DM in comparison to White American adults (Araneta 2019; Center for Disease Control and Prevention n.d.). Due to the complexity of T2DM, self‐management is integral to living with diabetes. Diabetes self‐management is characterised by seven key areas of care: healthy eating, physical activity, medication, blood sugar level, complication risk, emotional well‐being, and problem‐solving (Powers et al. 2015).Factors such as interpersonal relationships, access to healthcare, time, finances, language barriers, and, most notably, culture have been observed to influence self‐management (BeLue et al. 2012; Hallgren et al. 2015).

Historical and cultural factors are important considerations in disease and self‐management. Because Filipino culture is rooted in colonial history, there is a critical need to examine the role of colonialism on T2DM self‐management. Minoritized groups consider feelings of powerlessness as a barrier to managing T2DM (Jones and Crowe 2017). Prior studies that included Filipino Americans identified aspects of Filipino culture as central to diabetes self‐management, such as religion, stigma, food and family (Finucane and McMullen 2008). Catholicism, a significant influence from the Spanish colonisation, contributes to the belief that diseases are spiritual, treatable by prayer, and beyond the individual's control (“*bahala na*”) (Finucane and McMullen 2008; Sonsona 2014). Some Filipinos feel shameful (“*hiya*”) or guilty about having diabetes, as this may be considered a burden to the family (Finucane and McMullen 2008). Being advised to change one's diet may be met with reluctance due to the social and cultural implications of food for Filipinos (Finucane and McMullen 2008; Tolentino and Byrnes 2024). Self‐management is further complicated by the Filipino culture of prioritising the family over oneself (Finucane and McMullen 2008).

### 2.3 Colonial Mentality and Type 2 Diabetes in Filipino Americans

The Filipino American experience of health is shaped by racism and colonialism (Sabado‐Liwag et al. 2022). Despite evidence linking internalised racism to psychological and physical harms among minoritised individuals, it is unclear how colonial mentality influences chronic disease management (Office of the Surgeon General (US) et al. 2001; Smedley et al. 2003). Although prior studies have examined the relationship between colonial mentality and mental health (David and Nadal 2013), no studies have explored how colonial mentality affects self‐management of chronic diseases like T2DM, particularly among Filipino Americans. In addition, many intervention studies on self‐management do not account for the prevalence of colonial mentality experiences in Filipino Americans with T2DM (Finucane and McMullen 2008; Bender et al. 2018). Typical intervention studies focus on wellness factors such as diet and exercise; however, considering racism and colonialism as determinants of health is often overlooked (Forde et al. 2020).

Decolonizing health research requires assessing the effects of colonisation on marginalised individuals. We posit an association between colonial mentality (and its manifestations) and diabetes self‐management. As we do not currently understand the relationship between colonial mentality and diabetes self‐management, exploring this relationship is an important step. With the legacies of colonialism continuing to influence how Filipino Americans think, feel, and act, it is critical to understand self‐management within the context of colonial mentality.

## 3 The Study

### 3.1 Theoretical Framework

**FIGURE 1:**
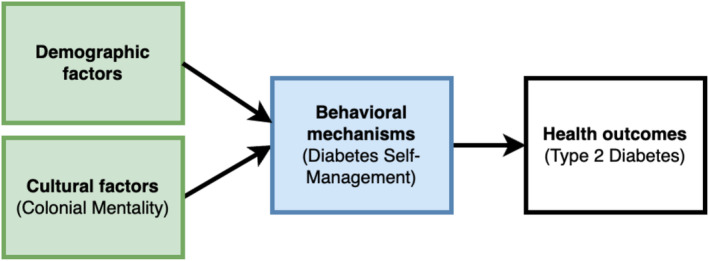
The theoretical framework adopted from McDade's general conceptual model for incorporating biological data into population‐based research.

We reformulated (Reed and Shearer 2023) McDade et al.'s conceptual model for incorporating biological data into population‐based research (McDade et al. 2007), investigating the dynamic associations between contextual factors and health (Figure 1). This framework emerged from diverse theoretical and disciplinary perspectives that inform the dynamic associations of context, physiology, behaviour, and health. Specifically, we used the behavioural mechanisms pathway of the framework, wherein the authors posit that some form of behavioural mechanisms mediates cultural factors and health outcomes. Colonial mentality was used to operationalise cultural factors, while diabetes self‐management was used for behavioural mechanisms.

### 3.2 Aim

The purpose of this study was to explore the relationship between colonial mentality and diabetes self‐management among Filipino Americans with T2DM.

## 4 Methodology

### 4.1 Design

This was a cross‐sectional study on the association between colonial mentality and self‐management of Filipino Americans with T2DM. The study followed the *Strengthening the Reporting of Observational Studies in Epidemiology* (STROBE) checklist for cross‐sectional studies (Cuschieri 2019).

### 4.2 Study Setting

The study was conducted in the United States, and we used an online survey platform, Qualtrics, to administer the questionnaire.

### 4.3 Participants/Data Collection

A sample of Filipino Americans with T2DM was invited to participate in our anonymous online survey. The inclusion criteria were participants of Filipino descent residing in the United States or self‐identifying as Filipino American, aged 18 years or older, proficient in English, and diagnosed with type 2 diabetes. Exclusion criteria were individuals not of Filipino American background and those diagnosed with type 1 diabetes or gestational diabetes. Participants were recruited through various modalities, including social media platforms such as Instagram and LinkedIn, in addition to engagement with national Filipino American organisations, including but not limited to the Philippine Nurses Association of America, UCLA's *Samahang Pilipino*, and Filipinx/a/o Community Health Association. These organisations were approached through email or direct messaging on their social media accounts, and a recruitment flyer was provided for their dissemination. The flyer contained a study overview, a link to the Qualtrics survey, and a Quick Response (QR) code for easy access. Data collection took over 17 months, between May 20, 2022, and October 31, 2023.

### 4.4 Variables

#### 4.4.1 Data Source/Measurement

##### 4.4.1.1 Health and Demographic Characteristics

Participants were asked to complete basic sociodemographic data, including age, gender, educational background, place of birth, duration of residence in the United States (if not born in the US), occupation, years since diabetes diagnosis, and any coexisting health conditions.

##### 4.4.1.2 Colonial Mentality

We used the Colonial Mentality Scale for Filipino Americans (CMS‐FA) to measure colonial mentality (David and Okazaki 2006). This instrument has 36 items with five subscales: (1) within‐group discrimination, (2) physical characteristics, (3) colonial debt, (4) cultural shame and embarrassment and (5) internalised cultural/ethnic inferiority. Participants rated each item on a scale ranging from 1 (strongly disagree) to 6 (strongly agree). To determine the presence of colonial mentality manifestations, a mean score of at least 3.5 on any of the Colonial Mentality Scale subscales was used as the threshold. Permission to use this instrument was obtained from the author. The reliability coefficients for the subscales range from *α* = 0.705 (colonial debt) to 0.927 (physical characteristics). The overall reliability coefficient was 0.968.

##### 4.4.1.3 Diabetes Self‐Management

Diabetes self‐management was measured using the 16‐item Diabetes Self‐Management Questionnaire (DSMQ) (Schmitt et al. 2013). The scale consists of 16 items distributed into four subscales: healthcare use, glucose control, diet control, and physical activity. Item responses are on a Likert four‐point scale with the response options ‘applies to me very much’ (3 points), ‘applies to me to a considerable degree’ (2 points), ‘applies to me to some degree’ (1 point), and ‘does not apply to me’ (0 points). Higher scores indicate more optimal self‐care. The scale has a long history of reliability and validity testing and offers valuable insights into diabetes self‐care and specific self‐care activities that impact glycemic control. The DSMQ was designed to facilitate scientific inquiries into psychosocial barriers affecting self‐care and glycemic control. The instrument is accessible under a Creative Commons Licence. The overall reliability score, as measured by Cronbach's alpha (*α* = 0.793), was based on 16 items, demonstrating a strong degree of internal consistency in the study's measurements.

### 4.5 Bias

Our study adopted a cross‐sectional design to investigate the association between diabetes self‐management behaviours and colonial mentality. To ensure the robustness of our findings, we utilised established measurement tools, specifically the Diabetes Self‐Management Questionnaire and the Colonial Mentality Scale, which have demonstrated strong reliability and validity, thereby reducing measurement bias. However, it is crucial to note that the cross‐sectional design limits our ability to establish causal relationships, necessitating caution in drawing causal inferences. We recognise the importance of ethical considerations, especially in measuring the sensitive concept of colonial mentality, including informed consent and ensuring participant well‐being.

### 4.6 Study Size

Using G*Power (Faul et al. 2007), we chose to include 34 participants in order to have 80% power to detect a medium effect size (*ρ*) of 0.4 with a significance level of 0.05 (two‐tailed test).

### 4.7 Statistical Analysis

Quantitative survey data were analysed descriptively using measures of central tendency (means, medians) and variability (SD, IQR, and frequencies). Bivariate correlation using Pearson's *r* was used to analyse the relationships of the variables with 95% CI. We tested assumptions for correlation, including linearity and testing for outliers. We also ran an independent *t*‐test to examine if there was a significant difference between genders and colonial mentality/colonial mentality manifestations. Non‐binary results for gender have been omitted in the non‐parametric analysis due to the small sample size (*n* = 1). Homogeneity of assumption was tested using Levene's test, and Welch's *t*‐test was reported for applicable results with violations of homogeneity of variance. All statistical testing used a significance level of *p* < 0.05. SPSS version 17 (IBM Corp, Armonk, NY) was used to analyse quantitative data.

### 4.8 Missing Data

We used SPSS Missing Value Analysis to examine missing data. For the main variables in this study (colonial mentality and diabetes self‐management), missing data ranged from 0% to 2.9%. Since missing data is less than 5%, we used list wise deletion to address missing data (Allison 2001).

### 4.9 Ethical Considerations

Ethical approval for this study was initially obtained from the University of Michigan Institutional Review Board (IRB; Protocol ID# HUM00215472; Approval date: 4/14/2022), and IRB oversight was transferred to UCLA IRB (Protocol ID# 22‐000650; Approval date: 8/3/2022) after the senior author changed institutions. The study was deemed exempt. Information about the study's purpose and risks was provided. Before the start of the survey, all eligible participants were presented with a comprehensive Informed Consent Form and were asked to consent to participate in the study. A small compensation (gift card) was provided to participants who completed the study.

## 5 Results

### 5.1 Sample Characteristics

A total of 322 participants accessed the survey, and 37 surveys were completed, representing an 11.5% response rate. On average, respondents were middle‐aged (M = 47.19 years old, SD = 17.61), had been diagnosed with T2D for 8.33 years (SD = 8.27), were married or in a domestic partnership (68.8%), were mostly Catholic (65.4%), and almost half were male (48.6%), with close to three‐fourths having at least an associate's degree (73%), and 61.2% reported an income of $75K or above annually. Other sample characteristics are displayed in Table 1.

**TABLE 1:** Study participant characteristics (*N* = 37).

Characteristic	*n* (%) for categorial variables M (SD) for continuous variables
Education	
Associates or technical degree	3 (9.4%)
Bachelor's degree	14 (43.7%)
Graduate or professional degree	6 (18.8%)
High school or general education diploma	2 (6.3%)
Some college, but no degree	7 (21.9%)
Marital status	
Divorced	1 (3.1%)
Married/domestic partner	22 (68.8%)
Single (never married)	8 (25.0%)
Widowed	1 (3.1%)
Income (annual)	
< $25,000	4 (12.9%)
$50–74K	5 (16.1%)
$75–99K	9 (29.0%)
$100–149K	11 (35.5%)
> $150K	2 (6.5%)
Religion	
Atheist	2 (7.7%)
Buddhist	1 (3.8%)
Catholic	17 (65.4%)
Christian—non‐Catholic	4 (15.4%)
Islam	2 (5.7%)
Employment	
Working	25 (78.1%)
Unemployed	1 (3.1%)
Retired	6 (18.8%)
Comorbidities	
Hypertension	15
Hyperlipidemia	9
Other cardiovascular disease	6
Overweight/obese	10
Chronic kidney disease	3
Family history of diabetes (any type)	
Yes	25 (71.4%)
No	8 (22.9%)
Unsure	2 (5.7%)
Birthplace	
Philippines	27 (77.1%)
USA	8 (22.9%)
Gender	
Female	14 (40.0%)
Male	17 (48.6%)
Non‐binary	1 (2.9%)
Years with diabetes	M = 8.33 (SD = 8.27)
Most recent HbA1c	M = 7.31 (SD = 1.72)
Duration living in the United States (for those born outside of the United States; years.)	M = 23.88 (SD = 2.78)
Age (years)	M = 47.19 (SD = 17.60)

### 5.2 Colonial Mentality by Gender

Male participants reported a higher overall colonial mentality mean (M = 3.27, SD = 1.14) or median scores (Mdn = 3.67 IQR = 1.90) compared to females (M = 2.99, SD = 1.14; Mdn = 2.93, IQR = 1.45). Similarly, all CM manifestations were higher in males than in females, as reported in Table 2. However, an independent t‐test revealed no statistically significant difference in colonial mentality scores between females and males.

**TABLE 2:** Independent *t*‐test comparing colonial mentality between females and males.

Colonial mentality/manifestations	Females		Males		*t*	df	*p*
	M	SD	M	SD			
Colonial Mentality score (overall)	2.99	0.85	3.27	1.14	−0.781	29	0.220
Cultural inferiority	2.61	1.04	3.01	1.27	−0.940	29	0.178
Within group discrimination	3.05	0.94	3.32	1.23	−0.675	29	0.253
Physical characteristics	3.05	0.94	3.25	1.37	−0.490^a^	28.132	0.628
Colonial debt	3.38	0.67	3.71	0.71	−1.317	29	0.099
Cultural shame	2.57	1.41	2.87	1.57	−0.553	29	0.292

^a^ Welch's *t* is reported due to a violation of homogeneity of variance.

### 5.3 Correlations

Table 3 shows the variables with statistically significant correlations between colonial mentality, diabetes self‐management, and sociodemographic data. A complete correlation matrix could be found in the Supplemental File (See Supporting Information—Data S1). Of the five manifestations of colonial mentality, internalised cultural/ethnic inferiority, cultural shame, and within‐group discrimination were all negatively associated with some form of diabetes self‐management activity. Out of all the sociodemographic variables, years living in the United States and years living with diabetes were negatively associated with four colonial mentality manifestations and the overall colonial mentality score.

**TABLE 3:** Select correlations between variables in the data, with a 95% Confidence Interval.

	IC/EI (95% CI)	CS (95% CI)	PC (95% CI)	WGD (95% CI)	CM (95% CI)
GLU	−0.39* (−0.64, 0.06)	−0.38* (−0.64, −0.05)	−0.27 (−0.56, 0.08) (NS)	−0.26 (−0.55, 0.08) (NS)	−0.29 (−0.57, 0.05) (NS)
HC	−0.39* (−0.64, 0.06)	−0.54** (−0.74, −0.25)	−0.31 (−0.59, 0.03) (NS)	−0.34* (−0.61, −0.01)	−0.39* (−0.64, −0.06)
DSMQ	−0.42* (−0.67, 0.10)	−0.44** (−0.66, −0.12)	−0.32 (−0.59, 0.02) (NS)	−0.29 (−0.57, 0.06)	−0.33 (−0.60, 0.06) (NS)
YRS US	−0.59** (−0.80, 0.26)	−0.65** (−0.83, −0.35)	−0.52** (−0.76, −0.17)	−0.55** (−0.77, −0.21)	−0.56** (−0.78, −0.22)
YRS DIAB	−0.47** (−0.79, 0.16)	−0.46** (−0.65, −0.04)	−0.37* (−0.63, −0.04)	−0.34* (−0.61, −0.01)	−0.39* (−0.61, −0.06)
HbA1c	0.09 (−0.26, 0.42) (NS)	0.07 (−0.28, 0.40) (NS)	−0.01 (−0.35, 0.34) (NS)	0.09 (−0.26, 0.42) (NS)	0.08 (−0.26, 0.42) (NS)

Abbreviations: CM, overall colonial mentality score; CS, cultural shame; DSMQ, diabetes self‐management questionnaire overall score; GLU, glucose control; HC, healthcare use; IC/EI, internalised cultural/ethnic inferiority; NS, not statistically significant at alpha level of < 0.05; PC, physical characteristics; WGD, within‐group discrimination; YRS DIAB, years living with diabetes; YRS US, years living in the United States.

* *p* < 0.05.

** *p* < 0.01.

#### 5.3.1 Cultural Shame, Internalised Cultural/Ethnic Inferiority, and Self‐Management (Glucose Control, Overall Self‐Management and Healthcare Use)

Internalised cultural/ethnic inferiority was negatively correlated to glucose control (*r* = −0.39, *p* = 0.024), healthcare use (*r* = −0.39, *p* = 0.024), and overall diabetes self‐management (*r* = −0.42, *p* = 0.013). Similarly, cultural shame was negatively correlated to glucose control (*r* = −0.38, *p* = 0.027), healthcare use (*r* = −0.54, *p* < 0.001), and overall diabetes self‐management (*r* = −0.44, *p* = 0.010). There were no statistically significant associations with other manifestations of colonial mentality and self‐management behaviours.

#### 5.3.2 Within‐Group Discrimination, Overall CM and Self‐Management (Healthcare Use)

Within‐group discrimination was negatively correlated with healthcare use (*r* = −0.34, *p* = 0.047), but it was not significantly associated with other diabetes self‐management behaviours.

The overall colonial mentality score was negatively correlated to healthcare use (*r* = −0.39, *p* = 0.023).

#### 5.3.3 Years Living in the United States and Years Living With T2DM

There was a moderate to strong negative relationship between years of living in the United States and years living with T2DM with internalised cultural/ethnic inferiority, cultural shame, physical characteristics, within‐group discrimination, and overall colonial mentality score. Cultural shame had the strongest negative relationship with years living in the United States (*r* = −0.65, *p* < 0.001), while internalised cultural/ethnic inferiority had the strongest negative relationship with years living with diabetes (*r* = −0.59, *p* < 0.001).

### 5.4 Sensitivity Analysis

In a sensitivity analysis removing the outliers present in the DSMQ (healthcare use, diet, PA) and CMS (CD) subscales, we found that the correlations reported in Table 3 were still statistically significant, and the correlation coefficients of many of the variables increased.

## 6 Discussion

To our knowledge, this study is the first to examine the association between colonial mentality and diabetes self‐management behaviours in a sample of Filipino Americans. Psychological frameworks have conceptualised how colonised individuals internalised oppression regarding their self‐identity and culture, demonstrating an association with their psychological well‐being (David 2008; Cokley 2002; Comas‐Díaz et al. 1998). Colonised individuals often internalise negative attitudes, including feelings of inferiority and shame toward their ethnic group (Nünning and Nünning 2015). For Filipino Americans in this study, we found an association between two manifestations of colonial mentality, internalised cultural/ethnic inferiority and cultural shame, to several self‐management behaviours: glucose control, healthcare use, and overall self‐management. Cultural inferiority is when individuals view their own cultural or ethnic group as inferior to others, while cultural shame is the feeling of embarrassment or humiliation about one's culture or ethnic background. These two colonial mentality manifestations are inversely associated with glucose control, healthcare use, and overall diabetes self‐management. Individuals who experience higher levels of internalised cultural/ethnic inferiority tend to report poorer glucose control, less frequent utilisation of healthcare services, and challenges in diabetes self‐management practices.

Colonial mentality encompasses cognitive, psychological, and cultural dimensions. Cognitively, colonial mentality involves the internalisation of a denigrating view of Filipinos and their culture, leading to a belief that their own cultural or ethnic group is inferior (Tuazon et al. 2019; Decena 2014).As with internalised racism, which involves adopting negative beliefs and stereotypes about one's racial or ethnic group, colonial mentality operates as a cognitive process through the internalisation of systemic or social messages that devalue one's cultural identity. This internalisation fosters cognitive biases, such as selectively focusing on the perceived negative aspects of one's culture while diminishing a sense of cultural pride and belonging (Decena 2014). These biases can manifest in suboptimal health behaviours, including poor glucose control and reduced engagement with healthcare services, as individuals may undervalue culturally informed practices or experience diminished self‐efficacy in managing their diabetes.

Emotionally, colonial mentality is marked by feelings of cultural shame and embarrassment about one's culture or ethnic background (David and Nadal 2013; Tolentino 2023). This can manifest as a sense of shame (“*hiya*” in Filipino) or guilt about having diabetes, as it may be considered a burden to the family. It may create low self‐esteem and distress that may directly undermine self‐efficacy and motivation for health‐promoting behaviours. Addressing these feelings of shame about one's culture through supportive, culturally congruent interventions is essential for improving diabetes self‐care and overall well‐being.

Culturally, colonial mentality is characterised by a deferential attitude toward Western culture and the devaluation of Filipino culture, as well as the adoption of dominant cultural norms (David 2008). This may lead to rejecting culturally rooted health practices or dietary behaviours, even when these practices may support effective diabetes management. For instance, in a qualitative study on Filipino Americans' self‐management, participants favoured Western dietary patterns over culturally appropriate and health‐promoting foods, as they perceived the Western diet as superior. (Tolentino 2023) Many diabetes interventions do not account for the prevalence of colonial mentality and often focus on diet and exercise, overlooking colonial mentality as a determinant of health (Tolentino et al. 2022).

Prior research indicates that colonial mentality affects mental health and well‐being (Tuazon et al. 2019; David and Nadal 2013; Ferrera 2011; Martinez et al. 2020). Colonial mentality leads to negative mental health help‐seeking behaviours (Tuazon et al. 2019), negative sense of belongingness, pessimistic attitudes toward personal and group characteristics, and greater levels of depression symptoms (David 2008).Similar to Filipino Americans' reluctance toward formal mental help‐seeking (Martinez et al. 2020), we posit that Filipino Americans' experience of internalised oppression may be associated with suboptimal self‐management behaviours.

Due to the interlocking impact of colonialism and racism on health (Sabado‐Liwag et al. 2022), a more significant characterisation in marginalised colonised groups than understanding racism alone as an SDOH is understanding colonialism and its impact on health. Racism can be understood as the legacy of colonialism, and these two must not be separated (Office of the High Commissioner for Human Rights 2024). Colonialism has been linked in the literature to chronic conditions like cardiovascular disease through structural experiences of discrimination that intersect with various SDOHs and result in worsened inequalities in health outcomes (McGibbon et al. 2013). Other works have linked colonialism to diabetes in Indigenous people, with a specific connection to intergenerational trauma. Colonialism is a structure, not an event. Therefore, especially when attempting to understand the health of Filipino immigrants and colonised people alike, we must not think of colonialism as a historical event of the past (McGibbon et al. 2013). Understanding colonialism as a structure is critical for the appropriate representation of the cause of health inequity, especially in the Filipino American community.

This study also found a difference in colonial mentality scores between male and female participants. Although our analysis revealed a non‐statistically significant difference, it is important to note that male participants reported higher overall CM scores than their female counterparts. This difference suggests that gender may play a role in how individuals perceive and internalise colonial mentality, which aligns with broader discussions on gender dynamics and cultural identity (Torralbas Fernández and Calcerrada Gutierrez 2020). The machismo phenomenon could potentially explain the discrepancy in CM scores between male and female participants (Nuñez et al. 2016). This social display of manliness is characterised by heavily leaning into traditional gender norms and can promote unhealthy representations of gender. With regard to our study, it is possible that our male participants resonated with these social ideations of gender and were more likely to hold other unhealthy cultural depictions and, therefore, lower CM scores. Our findings can be explained by the colonial roots of the machismo phenomenon, where colonial rule is understood to have produced binary and contradictory understandings of masculinity, resulting in this fragmented understanding of masculine expression (Anderson 2020).

### 6.1 Limitations and Areas for Future Research

This was a cross‐sectional study using self‐reported data with a small sample size, so additional caution should be used when generalising the results of this study. The sample size, while relatively small, was determined to be adequate based on a priori power analysis. While we used non‐random convenience sampling to recruit participants, which may limit the generalisability of the findings, this study was designed as an exploratory investigation to provide foundational insights into the relationship between colonial mentality and diabetes self‐management among Filipino Americans. The sample may not be representative of Filipino Americans, and future studies using random sampling techniques are recommended to validate and expand upon these findings. Also, the response rate for this study was 11%, which may further introduce bias and limit the representativeness of the findings. Low responses are common challenges in survey‐based studies, particularly among underrepresented populations such as Filipino Americans, due to factors such as survey fatigue or cultural barriers to participation (Shiyab et al. 2023). While this limitation does not undermine the internal validity of the findings, it limits the external validity of the results. Future studies should consider strategies to improve response rates, such as piloting, remuneration, multiple recruitment methods, and personalised touch (e.g., sending personalised invitations), as suggested by Shayid and colleagues (Shiyab et al. 2023).

We also acknowledge the need for further reporting on control variables and covariates to address potentially important confounding factors, such as education and acculturation, to enhance the study's scientific rigor and interpretability.

These findings raise several questions for future research consideration. First, what societal and cultural factors might contribute to the gender disparities in colonial mentality scores? Exploring the implications of higher colonial mentality in the context of gender, self‐esteem, cultural pride, and mental health is crucial. Understanding the underlying influences, such as historical legacies, media representations, and societal expectations, could illuminate this phenomenon. Second, future investigations should use longitudinal designs to confirm the directionality between colonial mentality and self‐management. Third, as this study used self‐reported measures, physiological measures such as biomarkers should be considered, including examining the biobehavioral sequelae of colonial mentality to diabetes‐related outcomes. Fourth, we recommend that future studies incorporate qualitative studies, such as focus groups or in‐depth interviews, exploring how Filipino Americans perceive and experience the impact of colonial mentality on their health. This will provide a richer understanding of the cognitive, psychological, and cultural mechanisms involved, offering insights that could inform future interventions. Lastly, the results invite discussions on potential interventions or strategies to mitigate the impact of colonial mentality on diabetes self‐management. Addressing and fostering a more inclusive and culturally affirming environment and returning to *kapwa* (Sevillano et al. 2023) (shared identity or togetherness; the process of reconnecting to Filipino values and restoring pride in one's cultural identity) can be pivotal in promoting healthy self‐identities and reducing the influence of colonial mentality.

## 7 Implications for Practice and Conclusion

Results from this study highlight the need for nurses to become familiar with the concept of colonial mentality in diabetes management. Healthcare providers should consider that colonial mentality, rooted in colonialism, may be associated with challenges in diabetes self‐management behaviours. Future interventions could benefit from expanding their focus beyond diet and exercise to also address cognitive, psychological, and cultural factors potentially associated with colonial mentality. Nurses should engage in emancipatory knowing that recognises the critical impact of racism and colonialism on the health of individuals, families, and communities (Chinn et al. 2022). Emancipatory knowing includes asking questions about the barriers to health, including structural and institutional barriers such as racism and colonialism.

Racism has increasingly become recognised as a social determinant of health (Paradies et al. 2015). In many colonised communities, colonialism is also becoming an important social determinant of health. Fostering culturally affirming environments and promoting healthy self‐identities will be important for mitigating the influence of colonial mentality. Addressing the legacy of colonialism and its manifestations is crucial for improving diabetes care in Filipino Americans.

## Author Contributions

R.P.E.RIII., B.A., G.J.G.P., G.M.G.P., D.L., K.R.C. and D.A.T. made substantial contributions to conception and design, or acquisition of data, or analysis and interpretation of data. R.P.E.RIII., B.A., G.J.G.P., G.M.G.P., D.L., K.R.C. and D.A.T. involved in drafting the manuscript or revising it critically for important intellectual content. R.P.E.RIII., B.A., G.J.G.P., G.M.G.P., D.L., K.R.C. and D.A.T. gave final approval of the version to be published. Each author should have participated sufficiently in the work to take public responsibility for appropriate portions of the content. R.P.E.RIII., B.A., G.J.G.P., G.M.G.P., D.L., K.R.C. and D.A.T. agreed to be accountable for all aspects of the work in ensuring that questions related to the accuracy or integrity of any part of the work are appropriately investigated and resolved.

## Ethics Statement

Ethical approval for this study was initially obtained from the University of Michigan, and IRB oversight was transferred to UCLA. The study was deemed as exempt. All eligible participants were presented with a comprehensive Informed Consent Form and were asked to consent to participate in the study. Permission was obtained to use the Colonial Mentality Scale for Filipino Americans (CMS‐FA) from the author.

## Conflicts of Interest

The authors declare no conflicts of interest.

## Supporting information


Data S1.

## Data Availability

The data that support the findings of this study are available on request from the corresponding author. The data are not publicly available due to privacy or ethical restrictions.
