# Supplementary material for: Colonial Mentality and Diabetes Self‐Management in Filipino Americans
Source: Nurs Open. 2025 Feb 27;12(3):e70175. doi: 10.1002/nop2.70175 (PMC11868027; doi:10.1002/nop2.70175)
Supplement: Supplementary file 1 — Data S1. [file NOP2-12-e70175-s001.pdf]

Supplemental File: Correlation matrix between colonial mentality manifestations and self-management behaviors

Table 1: Correlation matrix

Correlations

		sub_PA	sub_Diet	sub_Gluc	sub_HCUse	DSQMscore2	II_MEAN	WGD_MEAN	PC_MEAN	CD_MEAN	CS_MEAN	Mean score for CM	Diabyrs	A1C	YearsUS	YearofBirth	Age
sub_PA	Pearson Correlation	--															
sub_Diet	Pearson Correlation	.558**	--														
	Sig. (2-tailed)	<.001															
sub_Gluc	Pearson Correlation	.438**	.382*	--													
	Sig. (2-tailed)	.010	.026														
sub_HCUse	Pearson Correlation	.400*	.189	.534**	--												
	Sig. (2-tailed)	.019	.285	.001													
DSQMscore2	Pearson Correlation	.806**	.657**	.823**	.682**	--											
	Sig. (2-tailed)	<.001	<.001	<.001	<.001												
II_MEAN	Pearson Correlation	-.306	-.056	-.386*	-.388*	-.424*	--										
	Sig. (2-tailed)	.079	.751	.024	.024	.013											
WGD_MEAN	Pearson Correlation	-.178	.029	-.262	-.343*	-.289	.839**	--									
	Sig. (2-tailed)	.313	.872	.134	.047	.098	<.001										
PC_MEAN	Pearson Correlation	-.201	-.022	-.270	-.314	-.321	.878**	.829**	--								
	Sig. (2-tailed)	.253	.901	.122	.071	.065	<.001	<.001									
CD_MEAN	Pearson Correlation	.097	.262	-.013	-.179	.028	.623**	.715**	.671**	--							
	Sig. (2-tailed)	.583	.134	.944	.312	.877	<.001	<.001	<.001								
CS_MEAN	Pearson Correlation	-.281	.013	-.380*	-.543**	-.436**	.840**	.870**	.811**	.628**	--						
	Sig. (2-tailed)	.107	.942	.027	<.001	.010	<.001	<.001	<.001	<.001							
Mean score for CM	Pearson Correlation	-.199	.037	-.293	-.388*	-.325	.920**	.955**	.931**	.783**	.921**	--					
	Sig. (2-tailed)	.259	.835	.093	.023	.061	<.001	<.001	<.001	<.001	<.001						
Diabyrs	Pearson Correlation	.020	-.267	.089	.114	.016	-.473**	-.343*	-.370*	-.145	-.455**	-.394*	--				
	Sig. (2-tailed)	.911	.134	.621	.526	.931	.005	.047	.031	.412	.007	.021					
A1C	Pearson Correlation	-.064	.092	-.134	-.103	-.101	.089	.090	-.005	.138	.071	.077	.031	--			
	Sig. (2-tailed)	.729	.616	.464	.575	.581	.621	.620	.979	.443	.695	.670	.864				
YearsUS	Pearson Correlation	-.019	-.236	.343	.302	.224	-.591**	-.550**	-.521**	-.196	-.650**	-.561**	.732**	.036	--		
	Sig. (2-tailed)	.928	.255	.093	.142	.283	.001	.004	.006	.338	<.001	.003	<.001	.867			
Age	Pearson Correlation	.008	-.061	-.080	.152	-.019	-.326	-.333	-.236	-.038	-.344	-.298	.644**	-.027	.695**	-1.000**	--
	Sig. (2-tailed)	.968	.746	.667	.415	.918	.074	.068	.201	.839	.058	.104	<.001	.886	<.001	.000	

Notes:
Self-Management Behaviors:
Sub_PA: Physical Activity subscale; **sub_Diet**: Diet subscale; **sub_Gluc**: glucose control subscale; **sub_HCUse**: Healthcare use subscale; **DSQMscore2**: Mean Diabetes self-management score;

Colonial Mentaliy:
II_MEAN: mean score for cultural inferiority; **WGD_MEAN**: mean score for within-group discrimination; **PC_MEAN**: mean score for physical characteristics; **CD_MEAN**: mean score for colonial debt; b: mean score for cultural shame; **Mean score for CM**: mean score for colonial mentality

Diabyrs: years with diabetes; **A1C**: latest HbA1c score (self-reported**); YearUS**: years living in the US

**. Correlation is significant at the 0.01 level (2-tailed).
*. Correlation is significant at the 0.05 level (2-tailed).
